# Supplementary material for: Mechanism of assembly, activation and lysine selection by the SIN3B histone deacetylase complex
Source: Nat Commun. 2023 May 3;14:2556. doi: 10.1038/s41467-023-38276-0 (PMC10156912; doi:10.1038/s41467-023-38276-0)
Supplement: Supplementary file 1 — Supplementary Information [file 41467_2023_38276_MOESM1_ESM.pdf]

Supplementary Information for

Mechanism of assembly, activation and lysine selection by the SIN3B histone deacetylase complex

Mandy S.M. Wan[#1], Reyhan Muhammad[#1], Marios G. Koliopoulos[1], Theodoros I. Roumeliotis[2], Jyoti S. Choudhary[2], and Claudio Alfieri[1*]

[#]Contributed equally

[1]Division of Structural Biology, Chester Beatty Laboratories, The Institute of Cancer Research, London, UK.

[2]Functional Proteomics, Chester Beatty Laboratories, Cancer Biology Division, The Institute of Cancer Research, London, UK.

[*]Corresponding author. Tel: +44 20715 35087; E-mail: claudio.alfieri@icr.ac.uk

Supplementary Figs. 1 to 9

Supplementary Table 1

Supplementary Figure 1

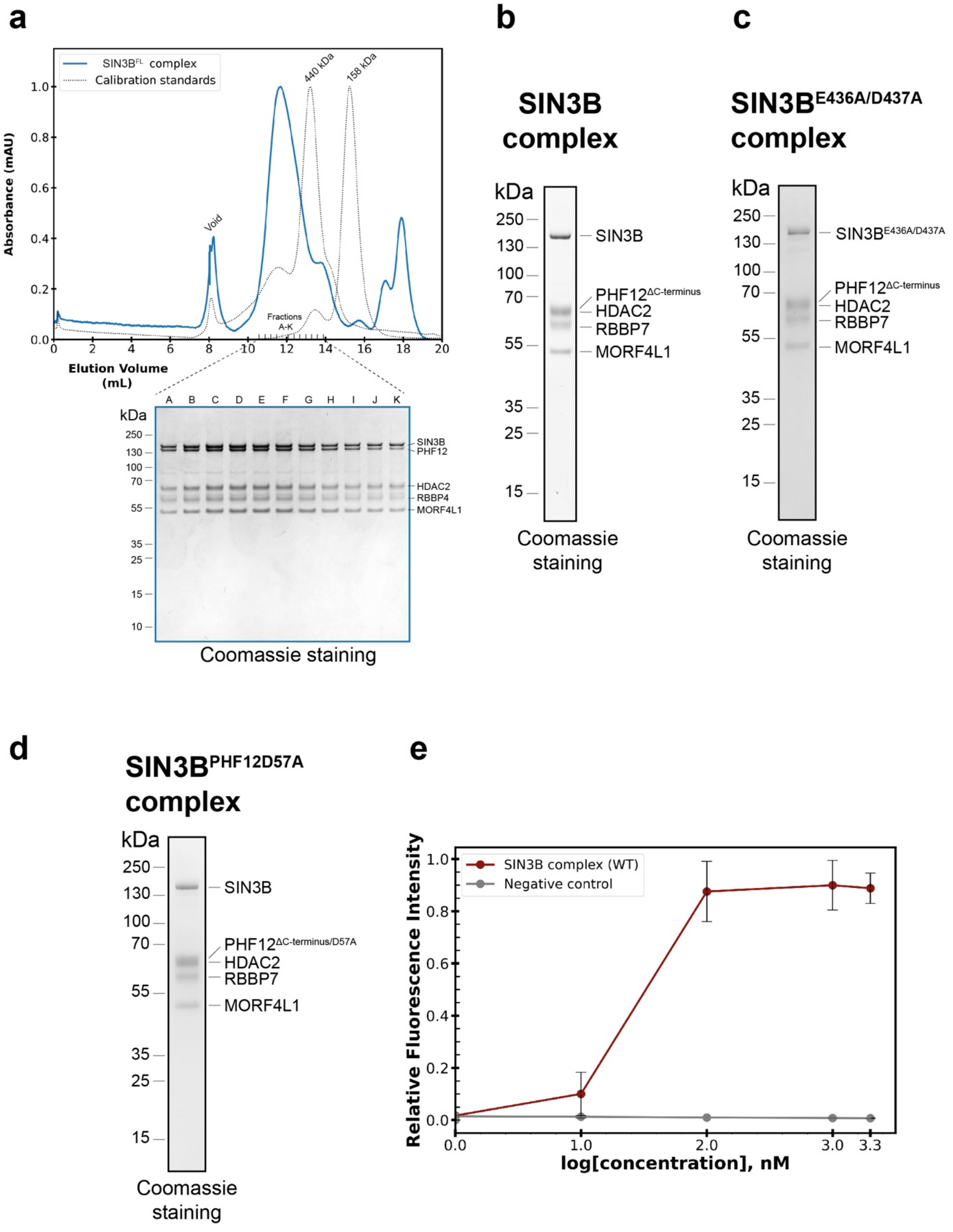

Supplementary Fig. 1. Biochemical reconstitutions of SIN3B complexes. a Size exclusion chromatography (SEC) chromatogram (Top) of the full length SIN3B complex (SIN3B^{FL}).

Calibration standards are illustrated for references of molecular weight. The SIN3B^{FL} complex elutes close to the 440 kDa molecular marker, the secondary peak eluting after the 158 kDa marker accounts for the 3C protease used to cleave the tags in SIN3B and MORF4L1 and the GST cleavage product. Coomassie stained gel of the eluted fractions (Bottom). **b-d** SDS PAGE of the protein complexes reconstituted in this study. The experiments in **(a-d)** were repeated independently three times with similar results. **e** HDAC assay showing that the SIN3B complex is active. Data are presented as mean values ± S.D. of independent experiments, n = 3.

Supplementary Figure 2

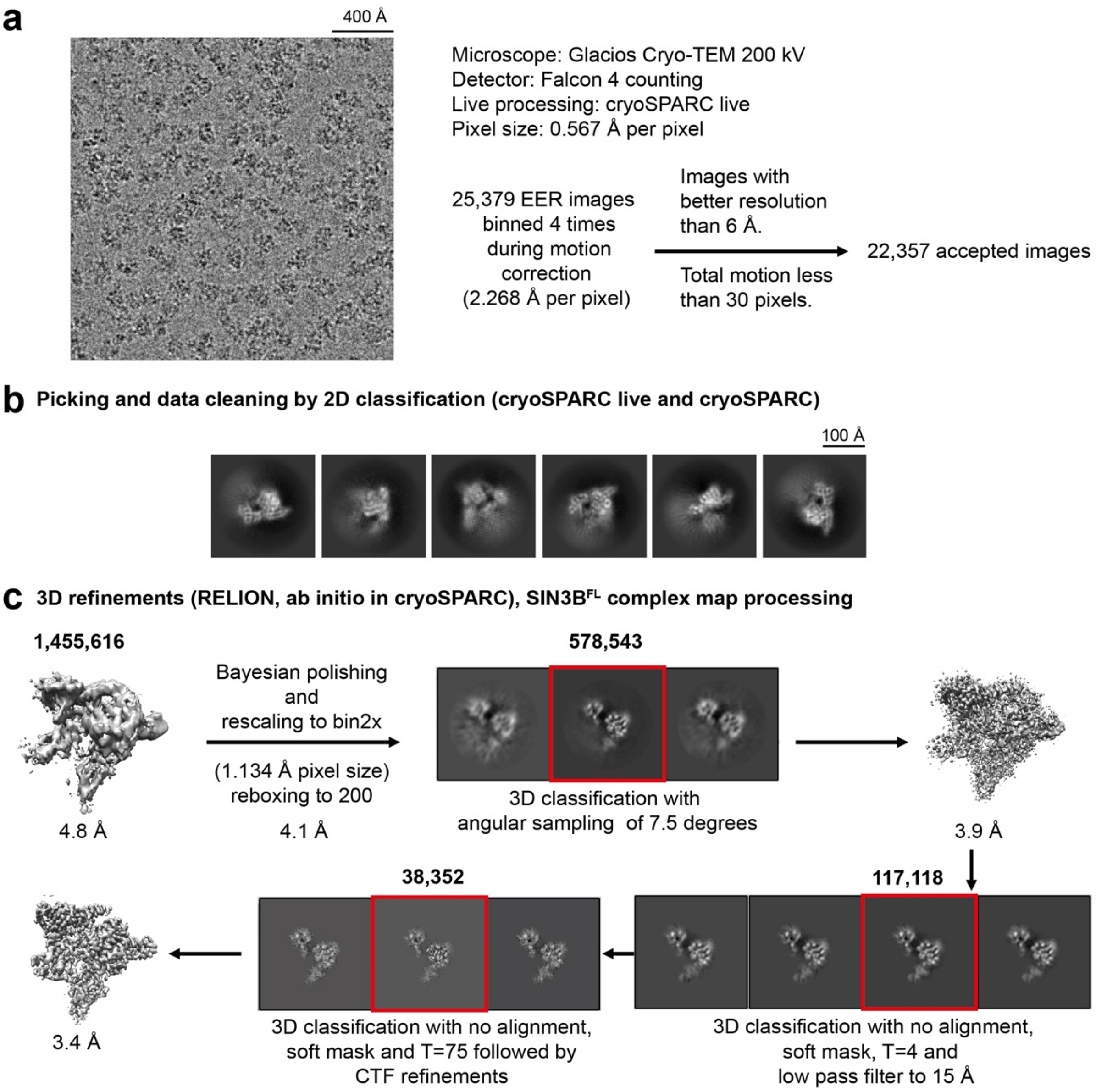

Supplementary Fig. 2. Cryo-EM analysis of the SIN3B full-length complex. Workflow showing a representative micrograph (low-pass filtered to 15 Å), the cryo-EM data collection

parameters (**a**) and the single-particle analysis pipeline for the SIN3B full-length ($SIN3B^{FL}$) complex (**b-c**). N. of particles at each classification step is indicated. More details are described in the main text and in the Methods section.

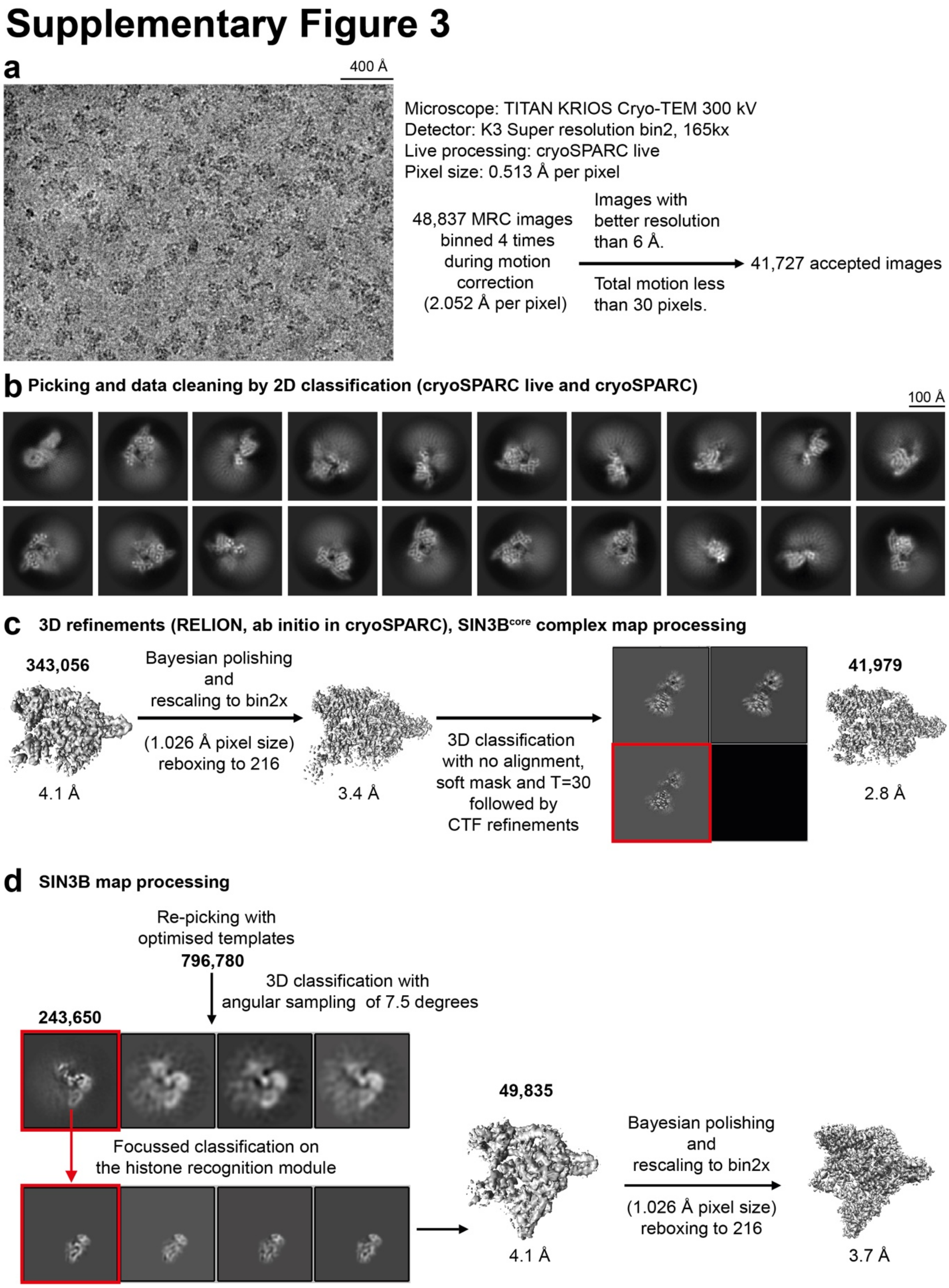

Supplementary Fig. 3. Cryo-EM analysis of the SIN3B complex. Workflow

showing a representative micrograph (low-pass filtered to 15 Å), the cryo-EM data collection parameters (**a**) and the single-particle analysis pipeline (**b-d**). N. of particles at each classification step is indicated. More details are described in the Methods section.

Supplementary Figure 4

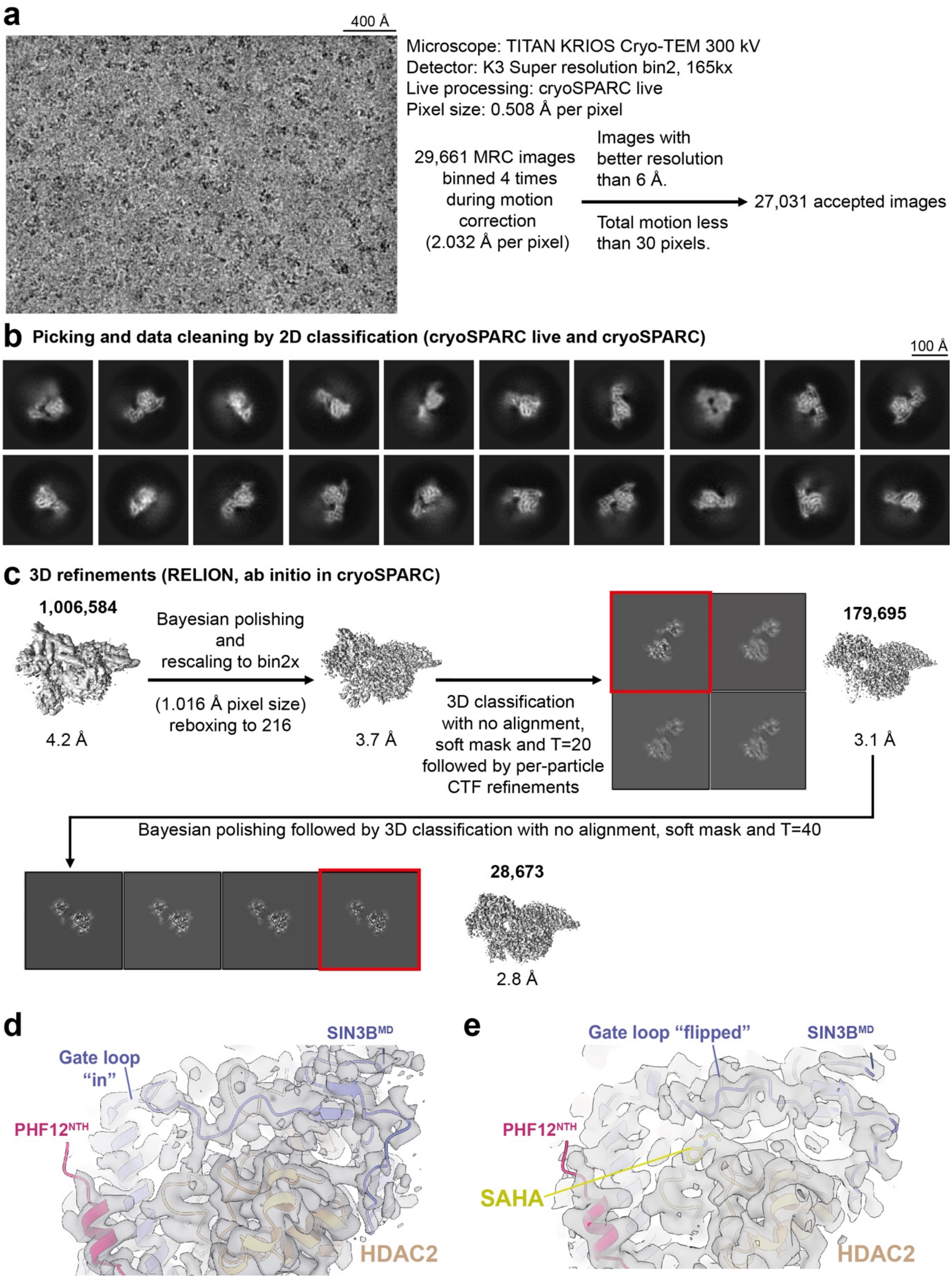

Supplementary Fig. 4. Cryo-EM analysis of the SIN3B^{core}:SAHA complex. Workflow showing a representative micrograph (low-pass filtered to 15 Å), the cryo-EM data collection parameters (**a**) and the single-particle analysis pipeline (**b-c**). N. of particles at each classification step is indicated. More details are described in the main text and in the Methods section. **d-e** Close-up view on the SIN3B$^{Gate\ loop}$ region from cryo-EM maps of the SIN3B^{core} complex (**d**), and the SIN3B^{core}:SAHA complex (**e**). Models coloured as in Fig. 1. SAHA in yellow.

Supplementary Figure 5

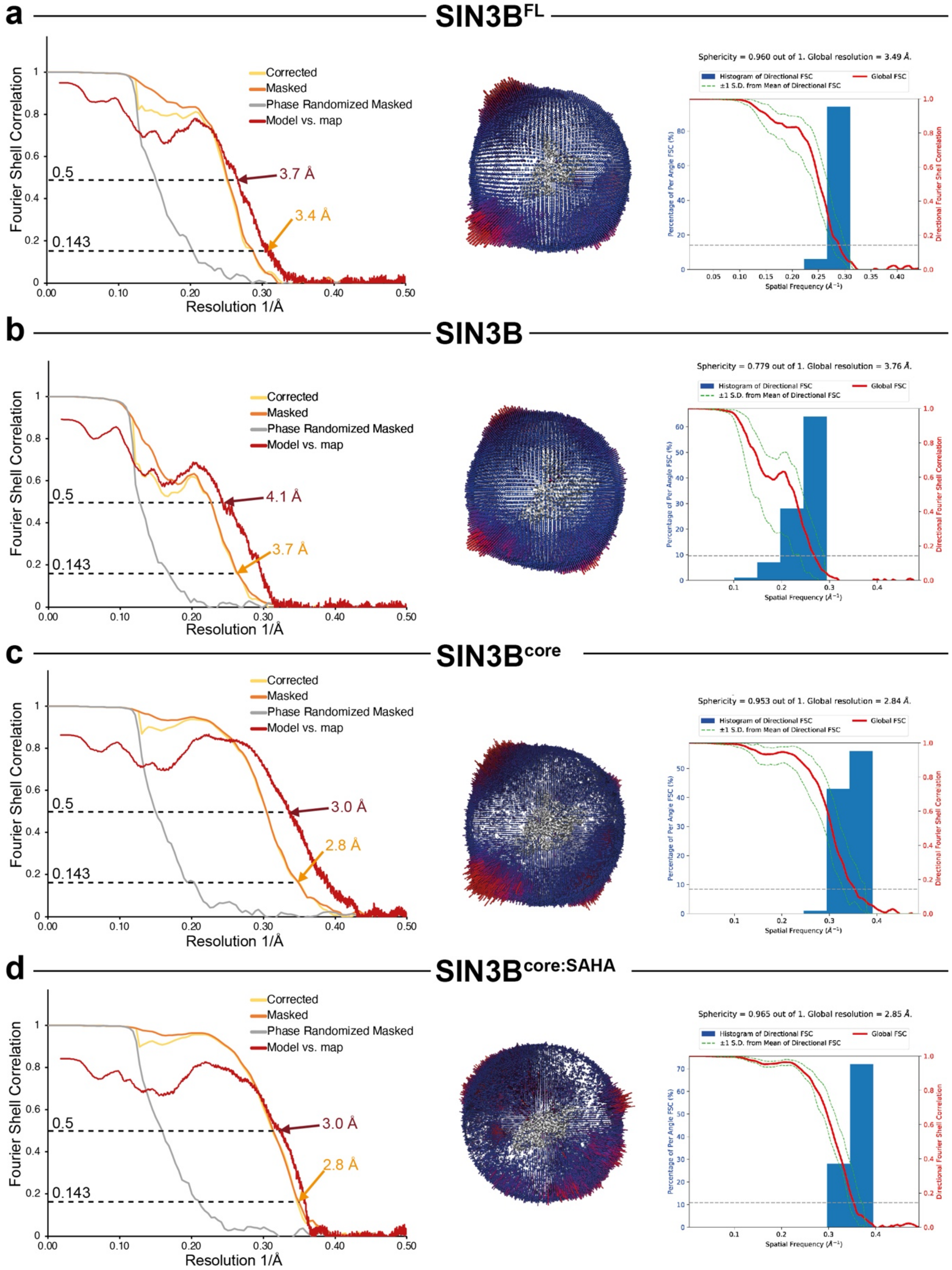

Supplementary Fig. 5. Cryo-EM analysis of the SIN3B complexes. a-d Fourier Shell Correlation (FSC) curves, angular distribution plot and plot of the directional FSC that represents a measure of directional resolution anisotropy for all the reconstructions are

shown. Directional FSC and sphericity determination was performed with the 3DFSC software.

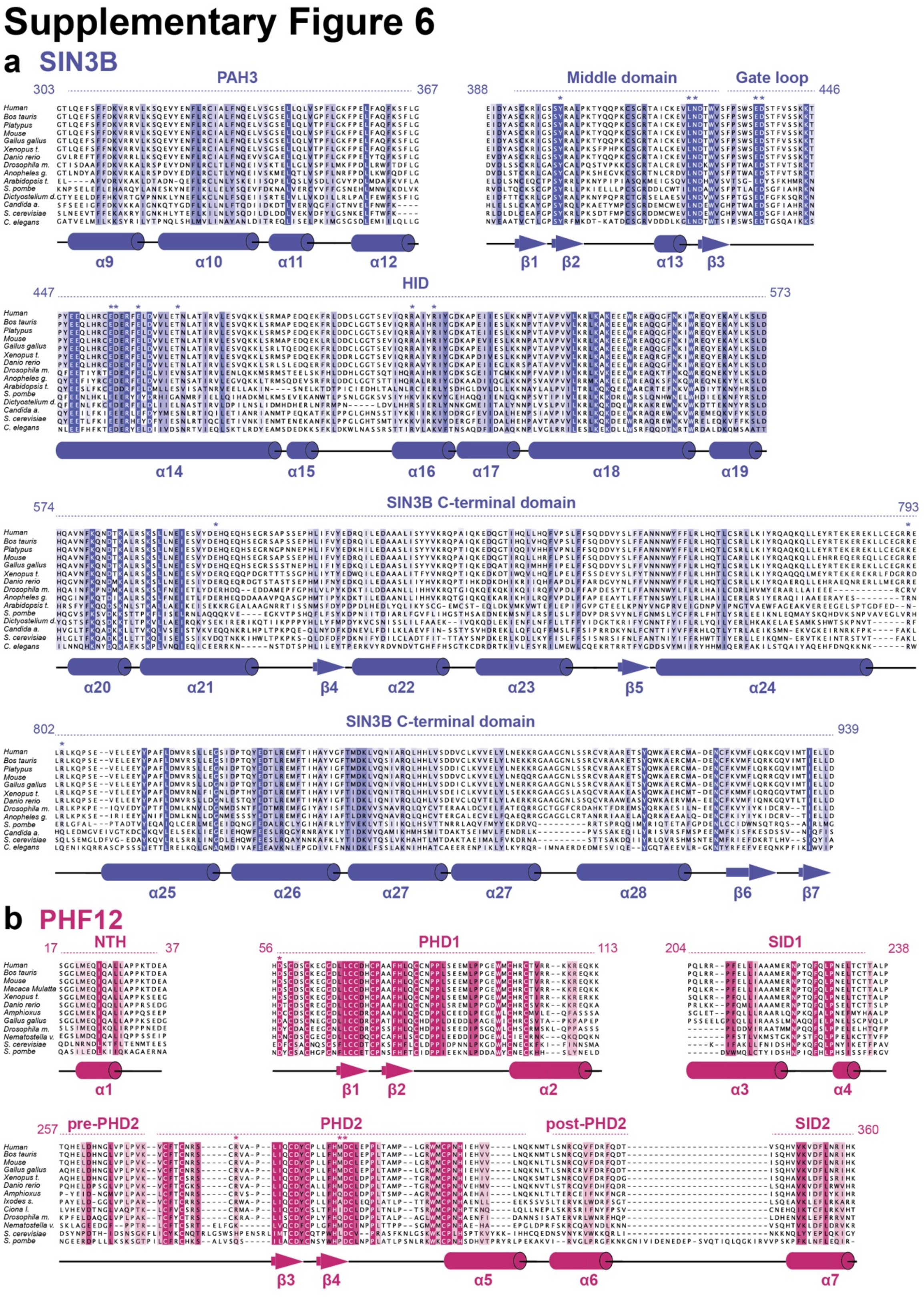

Supplementary Fig. 6. Conservation analysis on subunits of the SIN3B complex. a-b

Sequence alignments showing conservation of region of interest within this work. Residues which are mentioned in the main text are highlighted with an asterisk. Colour scheme for the SIN3B subunits as in Fig. 1.

Supplementary Figure 7

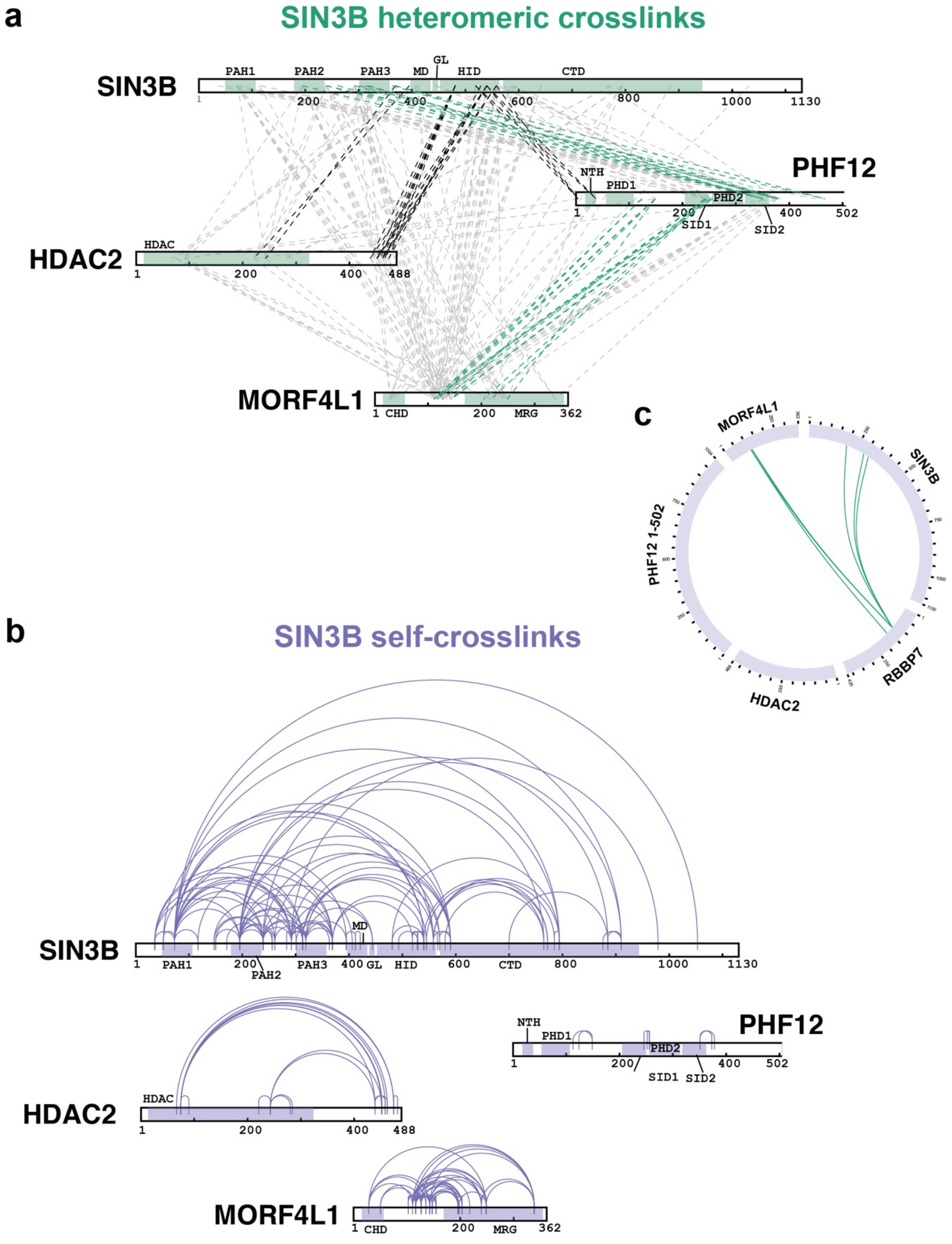

Supplementary Fig. 7. Representation of XL-MS results for the SIN3B apo complex. a Heteromeric crosslinks are visualised with xiVIEW (https://xiview.org/xiNET_website). SIN3B heteromeric crosslinks from the XL-MS data which are mentioned in the main text are highlighted in either black or green. Detected XLinked peptides are also shown in Supplementary Data 1. To obtain high confidence data for model interpretation and visualization, only the Xlinks to a value of >100 were used. **b** Self-crosslinks shown in the SIN3B subunits are shown with xiVIEW. **c** Heteromeric crosslinks between RBBP7 and the other SIN3B subunits are shown with xiVIEW.

Supplementary Figure 8

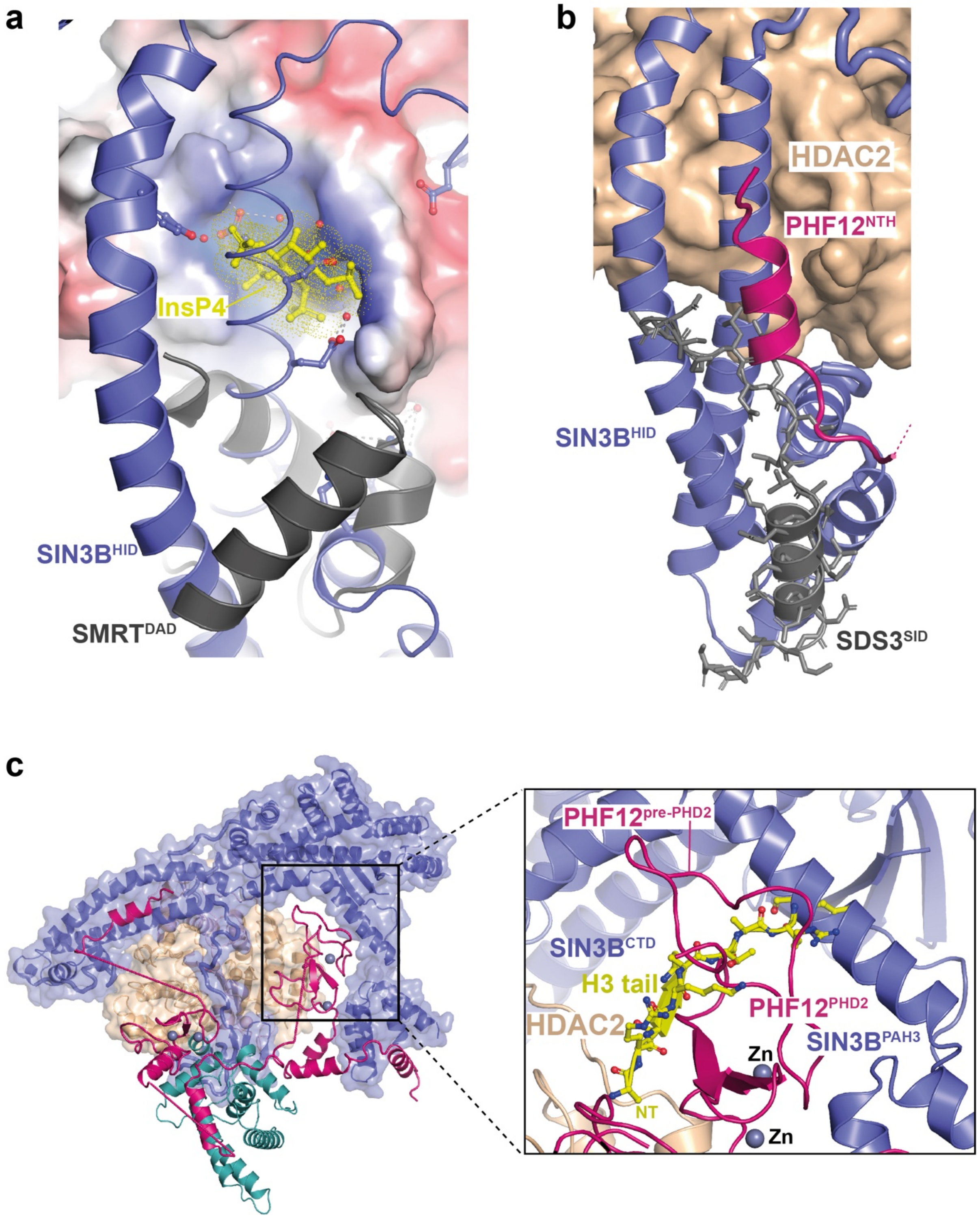

Supplementary Fig. 8. Structural comparisons of SIN3B substructures with related structures (part 1). a Superposition of the SMRT deacetylase activation domain (DAD, grey):HDAC3:Inositol tetraphosphate (InsP4, yellow) complex (PDB ID: 4A69) with our SIN3B complex structure (HDAC2 was the reference, shown as electrostatic potential surface). **b** Superposition of the SDS^{SID}:$SIN3A^{HID}$ structure (PDB ID: 2N2H) with our SIN3B

complex structure (SIN3B^{HID} as a reference). SDS^{SID} is in grey. **c** Modelling of the H3 histone tail based on the structure of BHC80^{PHD}:H3 (PDB ID: 2PUY) superposed to our SIN3B structure (PHD2 as a reference). H3 is in yellow. This docking exercise shows severe clashes between H3 N-terminal tail and SIN3B, and PHF12. SIN3B subunits from our structure are coloured as in Fig.1.

Supplementary Figure 9

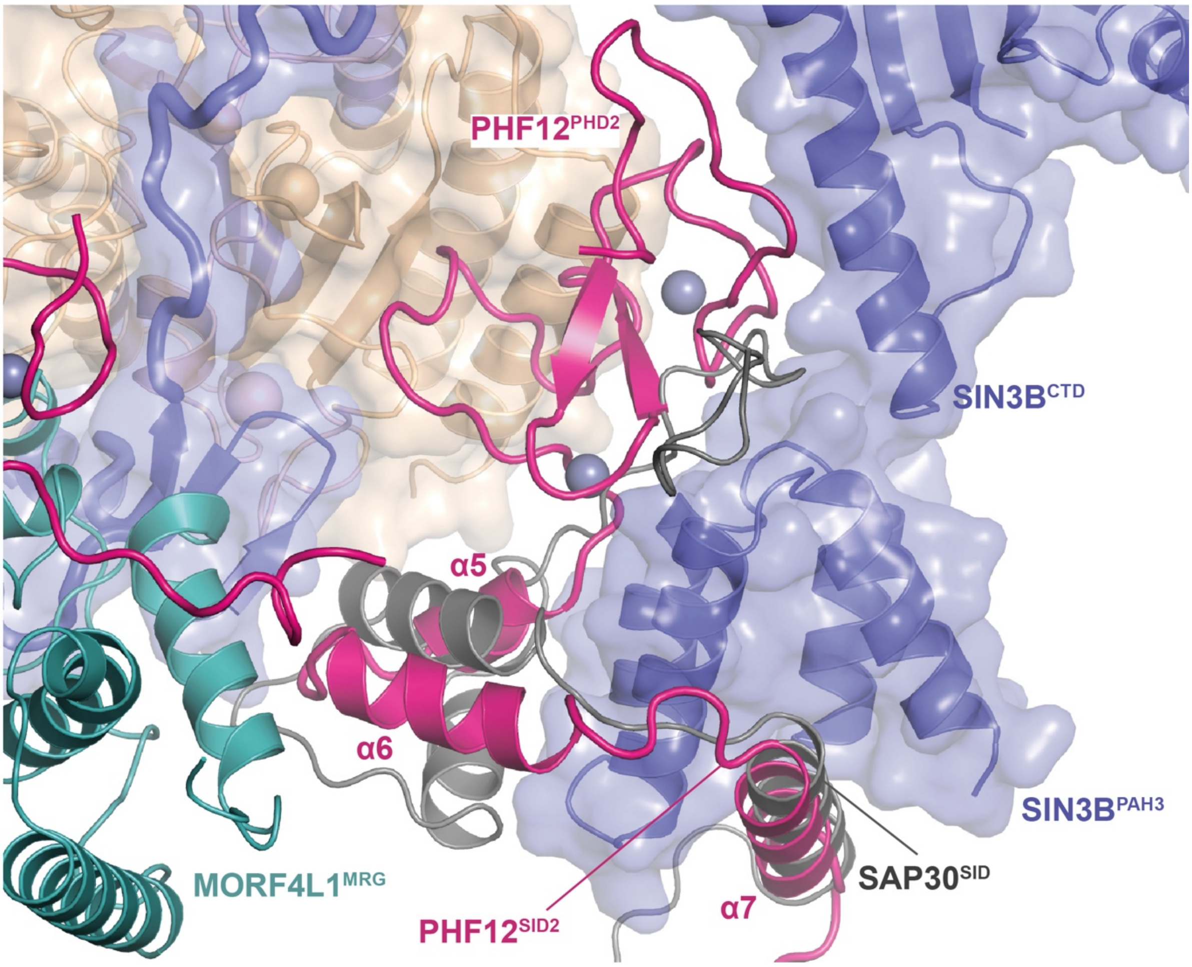

Supplementary Fig. 9. Structural comparisons of SIN3B substructures with related structures (part 2). Modelling of SAP30 onto the SIN3B structure based on the structure of SAP30^{SID}:SIN3A^{PAH3} structure (PDB ID: 2LD7) superposed to our SIN3B structure (SIN3B^{PAH3} as a reference). SIN3B subunits from our structure are coloured as in Fig.1.

Supplementary Table 1. Cryo-EM data collection, refinement and validation statistics

	SIN3B-FL	SIN3B	SIN3B-core	SIN3B-core:SAHA
**Data collection and processing**				
Magnification	240,000	165,000	165,000	165,000
Voltage (kV)	200	300	300	300
Electron exposure (e–/Å2)	60	60	60	60
Defocus range (μm)	-0.6 to -1.6	-0.6 to -1.6	-0.6 to -1.6	-0.6 to -1.6
Pixel size (Å)	0.567	0.513	0.513	0.508
Symmetry imposed	C1	C1	C1	C1
Initial particle images (no.)	1,455,616	796,780	343,056	1,006,584
Final particle images (no.)	38,352	49,835	41,979	28,673
Map resolution (Å)	3.4	3.7	2.8	2.8
FSC threshold	0.143	0.143	0.143	0.143
**Refinement**				
Initial model used (PDB code)	6XEC, 2LKM	6XEC, 2LKM	6XEC, 2LKM	6XEC, 2LKM
Model resolution (Å)	3.4	3.7	2.8	2.8
Map sharpening B factor (Å2)	-72	-97	-51	-41
Model composition				
Nonhydrogen atoms	10393	10393	10442	10491
Protein residues	1280	1280	1284	1284
Ligands	Zn(5), Ca(2)	Zn(5), Ca(2)	Zn(5), Ca(2), ACT(1)	Zn(5), Ca(2), SHH(1)
B factors (Å2)				
Protein (min/max/mean)	15.36/171.96/83.08	61.74/207.93/110.97	0.00/100.59/46.88	0.00/100.59/46.87
Ligand (min/max/mean)	93.87/216.36/142.03	83.96/215.77/137.10	0.00/131.51/50.19	0.00/131.51/33.84
R.m.s. deviations				
Bond lengths (Å)	0.004	0.005	0.003	0.004
Bond angles (°)	0.783	0.85	0.624	0.681
**Validation**				
MolProbity score	1.9	2.09	1.92	2
Clashscore	16.2	18.55	9.29	9.27
Poor rotamers (%)	0.7	1.15	2.46	2.64
Ramachandran plot				
Favored (%)	96.59	97.47	97.31	96.91
Allowed (%)	3.41	2.53	2.69	3.09
Disallowed (%)	0	0	0	0
