# Supplementary material for: Mechanism of assembly, activation and lysine selection by the SIN3B histone deacetylase complex
Source: Nat Commun. 2023 May 3;14:2556. doi: 10.1038/s41467-023-38276-0 (PMC10156912; doi:10.1038/s41467-023-38276-0)
Supplement: Supplementary file 3 — Description of Additional Supplementary Files [file 41467_2023_38276_MOESM3_ESM.pdf]

Description of Additional Supplementary Files

File Name: Supplementary Data 1
Description: Crosslinking mass spectrometry data.

File Name: Supplementary Movie 1
Description: Cryo-EM maps and models of the SIN3B complex structures. SIN3B full-length, SIN3B core, SIN3B:SAHA complex cryo-EM structures are presented.
