# Supplementary material for: Mechanism of assembly, activation and lysine selection by the SIN3B histone deacetylase complex
Source: Nat Commun. 2023 May 3;14:2556. doi: 10.1038/s41467-023-38276-0 (PMC10156912; doi:10.1038/s41467-023-38276-0)
Supplement: Supplementary file 6 — Reporting Summary [file 41467_2023_38276_MOESM6_ESM.pdf]

nature portfolio

Corresponding author(s): Claudio Alfieri

Last updated by author(s): Mar 30, 2023

Reporting Summary

Nature Portfolio wishes to improve the reproducibility of the work that we publish. This form provides structure for consistency and transparency in reporting. For further information on Nature Portfolio policies, see our Editorial Policies and the Editorial Policy Checklist.

Statistics

For all statistical analyses, confirm that the following items are present in the figure legend, table legend, main text, or Methods section.

n/a	Confirmed	
☐	☒	The exact sample size (*n*) for each experimental group/condition, given as a discrete number and unit of measurement
☐	☒	A statement on whether measurements were taken from distinct samples or whether the same sample was measured repeatedly
☐	☒	The statistical test(s) used AND whether they are one- or two-sided<br>*Only common tests should be described solely by name; describe more complex techniques in the Methods section.*
☐	☒	A description of all covariates tested
☐	☒	A description of any assumptions or corrections, such as tests of normality and adjustment for multiple comparisons
☐	☒	A full description of the statistical parameters including central tendency (e.g. means) or other basic estimates (e.g. regression coefficient) AND variation (e.g. standard deviation) or associated estimates of uncertainty (e.g. confidence intervals)
☐	☒	For null hypothesis testing, the test statistic (e.g. *F*, *t*, *r*) with confidence intervals, effect sizes, degrees of freedom and *P* value noted<br>*Give P values as exact values whenever suitable.*
☒	☐	For Bayesian analysis, information on the choice of priors and Markov chain Monte Carlo settings
☒	☐	For hierarchical and complex designs, identification of the appropriate level for tests and full reporting of outcomes
☒	☐	Estimates of effect sizes (e.g. Cohen's *d*, Pearson's *r*), indicating how they were calculated

Our web collection on statistics for biologists contains articles on many of the points above.

Software and code

Policy information about availability of computer code

Data collection	EPU 3 (Thermofisher), cryoSPARC live 3.2.0 (Ref.61)
Data analysis	cryoSPARC 3.2.0 (Ref.61), RELION 3.1.1 (Ref. 63), PHENIX 1.20.1 (Ref. 65), Coot 0.9.6 (Ref. 64), AlphaFold v2.1.0 (Ref. 51), Image J Fiji v2.1.0/1.53c, UCSF pyem v0.5 (https://doi.org/10.5281/zenodo.3576630).

For manuscripts utilizing custom algorithms or software that are central to the research but not yet described in published literature, software must be made available to editors and reviewers. We strongly encourage code deposition in a community repository (e.g. GitHub). See the Nature Portfolio guidelines for submitting code & software for further information.

Data

Policy information about availability of data

All manuscripts must include a data availability statement. This statement should provide the following information, where applicable:

- Accession codes, unique identifiers, or web links for publicly available datasets
- A description of any restrictions on data availability
- For clinical datasets or third party data, please ensure that the statement adheres to our policy

The structural coordinates have been deposited in the Protein Data Bank with the following accession numbers: PDB-ID 8C60, 8BPA, 8BPB, and 8BPC; the EM data have been deposited in the Electron Microscopy Data Bank with the following accession numbers: EMD-16449, EMD-16147, EMD-16148, and EMD-16149. The mass spectrometry proteomics data have been deposited to the ProteomeXchange Consortium via the PRIDE75 partner repository with the dataset identifier

PXD040006. All the other data and materials used for the analysis are available from the corresponding author upon reasonable request. Source data are provided with this paper.

Human research participants

Policy information about studies involving human research participants and Sex and Gender in Research.

Reporting on sex and gender	N.A.
Population characteristics	N.A.
Recruitment	N.A.
Ethics oversight	N.A.

Note that full information on the approval of the study protocol must also be provided in the manuscript.

Field-specific reporting

Please select the one below that is the best fit for your research. If you are not sure, read the appropriate sections before making your selection.

☒ Life sciences ☐ Behavioural & social sciences ☐ Ecological, evolutionary & environmental sciences

For a reference copy of the document with all sections, see nature.com/documents/nr-reporting-summary-flat.pdf

Life sciences study design

All studies must disclose on these points even when the disclosure is negative.

Sample size	No sample size calculation was performed. Biochemical experiments (Figure 2d-e, 3e-g, and 5e-h, and Supplementary Figure 1e) were performed in biological triplicates (at least n=3) in order to allow for calculating mean, standard error of the mean and performing unpaired t-tests and showing significance in the observed differences. The other experiments were still performed in biological triplicates (n=3) for assessing reproducibility.
Data exclusions	no data were excluded.
Replication	Biochemical experiments were repeated independently at least three times with similar results.
Randomization	N.A. (Randomization is not required in our study because it does not involve a clinical trial or a treatment allocation).
Blinding	N.A. (Blinding is not required in our study because it does not involve a clinical trial or a treatment allocation).

Reporting for specific materials, systems and methods

We require information from authors about some types of materials, experimental systems and methods used in many studies. Here, indicate whether each material, system or method listed is relevant to your study. If you are not sure if a list item applies to your research, read the appropriate section before selecting a response.

Materials & experimental systems

n/a	Involved in the study	
☐	☒	Antibodies
☐	☒	Eukaryotic cell lines
☒	☐	Palaeontology and archaeology
☒	☐	Animals and other organisms
☒	☐	Clinical data
☒	☐	Dual use research of concern

Methods

n/a	Involved in the study	
☒	☐	ChIP-seq
☒	☐	Flow cytometry
☒	☐	MRI-based neuroimaging

Antibodies

Antibodies used	H3K9ac, H3K27ac, H3K14ac and H4 primary antibodies (Cell Signalling #9649, #D5E4, Sigma-Aldrich #07-353, and Abcam #ab7311 respectively), goat-anti-rabbit-HRP conjugated secondary antibody (Abcam, #ab205718). All the primary antibodies were diluted

	1:2000. The secondary antibody was diluted 1:10,000.
Validation	H3K9ac, H3K27ac, H3K14ac and H4 primary antibodies (PMID 32501215). Goat-anti-rabbit-HRP conjugated secondary antibody (PMID: 33883691).

Eukaryotic cell lines

Policy information about cell lines and Sex and Gender in Research

Cell line source(s)	Insect cell lines for baculovirus expression: Spodoptera frugiperda Sf21 cells (SF9), Trichoplusia ni (Hi-five)
Authentication	Insect cells lines from Invitrogen were not further authenticated
Mycoplasma contamination	Protein expression cell lines were not tested for Mycoplasma contamination.
Commonly misidentified lines (See ICLAC register)	N.A.
